# Successor to Dr. Stockton

**Published:** 1887-08

**Authors:** 


### *SUCCESSOR TO DR. STOCKTON.*

At a recent meeting of the Faculty of the Medical Department of Niagara University, the vacancy in the Chair of Materia Medica and Therapeutics was filled by the promotion of Dr. F. R. Campbell, who for several years has held the important position as lecturer on hygiene in the college. During his connection with the college, he is said to have been most faithful in the discharge of his duty. His lectures have been characterized by a clearness and exactness rarely seen. The public estimation of Dr. Campbell is very high, as he has a reputation fairly won, without having resorted to spread-eagle advertisements, which the public, as well as the profession, so despise. The light in which he is regarded by the public is best shown by the following extracts from the Buffalo *Courier:*

The Chair of Materia Medica and Therapeutics was recently made vacant in the Medical Department of the Niagara University by the resignation of Dr. Charles G. Stockton, who has heretofore occupied the position with credit to himself and the school. After considering the names of several gentlemen of ability and experience, it has been decided to tender the position to Dr. F. R. Campbell, who has at times acted as assistant, as well as occupied the position of lecturer on hygiene in the school. It is believed that the doctor is in every way worthy of the honorable and important position to which he has been promoted, as he is a gentleman of excellent literary attainments, being a graduate in arts from Rochester University, and has had ample experience, derived from a large and rapidly-increasing medical practice. His professional standing in the community is high, and by his medical contributions, many of which have been of a very superior character and have found place in foreign journals, and by his translations of important articles from the French and Italian, with which languages he is familiar, he has won an extended reputation as a scholar and writer. As a teacher he has excelled, not so much in oratory, but by the clearness and succinctness with which he has presented subjects. Indeed, Dr. Campbell seems to be one of the rising medical men of Buffalo, and he will, without doubt, distinguish himself in the profession and in the school with which he has been identified as a lecturer from its organization, by his energy and scholarship. The appointment will commend itself to the medical profession and to the friends of the school.

### NIAGARA UNIVERSITY'S APPOINTMENT.

A meeting of the faculty of the medical department of Niagara University was held yesterday to fill the existing vacancy in the professorship of materia medica, which was caused by the resignation of Dr. Stockton, who accepted the head of the Buffalo University's faculty. The unanimous vote was given to Dr. Fred R. Campbell of this city, who is now a lecturer in the college on the subject of hygiene.

During the last few years he has attracted the attention of the profession in this city, and in fact of the surrounding country, by his articles in the BUFFALO MEDICAL AND SURGICAL JOURNAL, of which he is an associate editor. A recent number of the JOURNAL contains an article on the prevention of diarrhœal diseases in children, which is said to have been universally recommended by experts on these subjects. On this account, and because of his broad and liberal education, he was doubtless chosen. He is a native of this State, and was educated at the Lockport High School, and afterwards at the Rochester University, where he graduated with honor. His medical education was obtained in this city and the medical department of the University of New York. For some months he was clinical assistant at the Rochester Insane Asylum. His work as a sanitarian in this city has been thorough, and as a health inspector he has rendered great service to the city. This selection has been made after careful consideration of the qualities of many physicians who could have accepted the position. The doctor has as large a practice as any young physician in the city, and is greatly esteemed by his patients. It is felt that he will fill his new position with credit to himself and to the college. His nomination will be forwarded to the trustees at once.—*From Buffalo Express.*

As will be seen by these extracts, the Doctor has unexceptional educational advantages; has had a large practice since graduation, and is believed to be in every way qualified to fill this exalted position. This belief we share to the fullest extent, and congratulate the faculty of Niagara University on its admirable selection. The nomination will be sent to the Trustees at once for appointment. The course of lectures will begin with the term, September 21, 1887.

